# Effect of Metal Complexing on Mn–Fe/TS-1 Catalysts for Selective Catalytic Reduction of NO with NH_3_

**DOI:** 10.3390/molecules28073068

**Published:** 2023-03-29

**Authors:** Yuanyuan Ma, Wanting Liu, Zhifang Li, Yuhang Sun, Mingyuan Shi, Zheng Nan, Ruotong Song, Liying Wang, Jingqi Guan

**Affiliations:** 1 College of Chemistry and Chemical Engineering, Qiqihar University, Qiqihar 161006, China; 2 College of Materials Science and Engineering, Qiqihar University, Qiqihar 161006, China; 3 Institute of Physical Chemistry, College of Chemistry, Jilin University, 2519 Jiefang Road, Changchun 130021, China

**Keywords:** Mn-Fe/TS-1, one-pot synthesis, NH_3_-SCR, metal complexing, resistance to H_2_O and SO_2_

## Abstract

TS-1 zeolite with desirable pore structure, an abundance of acidic sites, and good thermal stability promising as a support for the selective catalytic reduction of NO with NH_3_ (NH_3_-SCR). Herein, a series of Mn–Fe/TS-1 catalysts have been synthesized, adopting tetraethylenepentamine (TEPA) as a metal complexing agent using the one-pot hydrothermal method. The introduced TEPA can not only increase the loading of active components but also prompts the formation of a hierarchical structure through decreasing the size of TS-1 nanocrystals to produce intercrystalline mesopores during the hydrothermal crystallization process. The optimized Mn–Fe/TS-1(R-2) catalyst shows remarkable NH_3_-SCR performance. Moreover, it exhibits excellent resistance to H_2_O and SO_2_ at low temperatures. The characterization results indicate that Mn–Fe/TS-1(R-2) possesses abundant surface Mn^4+^ and Fe^2+^ and chemisorbed oxygen, strong reducibility, and a high Brønsted acid amount. For comparison, Mn–Fe/TiO_2_ displays a narrower active temperature window due to its poor thermostability.

## 1. Introduction

Nitrogen oxides (NO*_x_*) are associated with a host of environmental issues, such as acid rain, haze, and photochemical smog, which severely endanger public health [1,2]. Selective catalytic reduction of nitrogen oxides with ammonia (NH_3_-SCR) is a crucial method to effectively control NO*_x_* emission [3,4]. Presently, the commercial V_2_O_5_–WO_3_/TiO_2_ (VWTi) catalyst is employed extensively for controlling NO*_x_* emissions. However, there are still many problems in the practical application of the VWTi catalyst, such as narrow temperature windows, poor high-temperature stability, the poisonousness of V_2_O_5_, and so on [5,6]. Accordingly, numerous studies have been devoted to exploiting vanadium-free NH_3_-SCR catalysts in recent years.

Among the frequently adopted V-free metal oxide catalysts, Mn-based catalysts have been proven to be remarkable low-temperature denitrification catalysts due to polyvalent oxidation and high redox capabilities [7,8]. However, the practical application of single-metal MnO*_x_* catalysts is restricted due to their narrow operating temperature window, and poor H_2_O/SO_2_ resistance [9,10]. Therefore, other transition metals/rare earth metal oxides serve as active components to perfect the Mn-based catalysts and improve the denitrification performance [11,12]. In recent years, Mn–Fe composite catalysts have gained extensive attention for their superior SCR activity and tolerance to SO_2_/H_2_O at low temperatures [13,14,15]. Li et al. [16] reported that Fe_2_O_3_–MnO_2_/TiO_2_ catalyst synthesized through a conventional impregnation method displayed excellent low-temperature activity in the wide temperature range of 100−325 °C and superior sulfur poisoning resistance. Additionally, Chen et al. [17] investigated La-modified TiO_2_ as the support of Fe–Mn/TiO_2_(*x* La) catalyst for NH_3_-SCR at low temperatures, revealing that La-modified Fe–Mn/TiO_2_(*x* La) catalyst enhanced SO_2_ resistance through an increase in Brønsted acid sites and accelerating the electron transfer between La and active components to restrain the adsorption and oxidation of SO_2_ on the catalyst. Nevertheless, TiO_2_ as a support in NH_3_-SCR possesses poor thermal stability and is still unsatisfactory for practical application [18].

It has been reported that silicon atoms replacing a small number of titanium atoms to form TS-1 zeolite with MFI structure can improve thermal stability and surface acidity [19]. Wang et al. [20] obtained MnO*_x_*–FeO*_x_*/TS-1 via a wet impregnation method, and the catalyst with the TS-1 support displayed superior denitration ability and H_2_O resistance due to the enhanced surface acidity and redox ability. Considering that NH_3_-SCR performance could be influenced by controlling the pore structure of the TS-1 support, a novel Fe−Mn/TS-1 catalyst with a micro-mesoporous structure was prepared using a one-pot hydrothermal synthesis method. The Fe−Mn/TS-1 catalyst showed excellent catalytic activity and H_2_O/SO_2_ resistance in a low-temperature SCR reaction. The influence of the additional quantity of metal complexing agent TEPA on NO*_x_* conversion has been investigated, and the SO_2_ and H_2_O resistance has also been explored.

## 2. Results and Discussion

### 2.1. XRD Patterns

**Figure 1:**
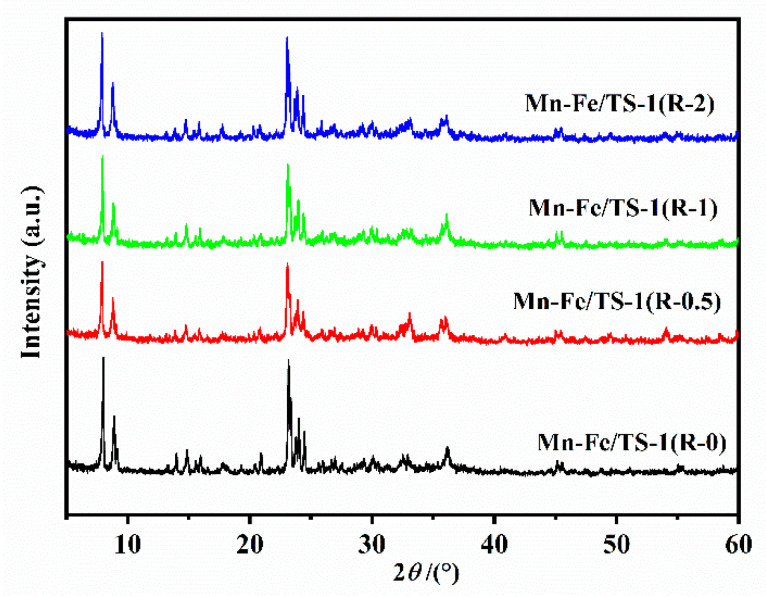
XRD patterns of Mn–Fe/TS-1(R-0), Mn–Fe/TS-1(R-0.5), Mn–Fe/TS-1(R-1), Mn–Fe/TS-1(R-2).

Figure 1 displays the XRD patterns of Mn–Fe/TS-1(R-0), Mn–Fe/TS-1(R-0.5), Mn–Fe/TS-1(R-1), and Mn–Fe/TS-1(R-2). The diffraction peaks at 7.9, 8.8, 23.1, 23.8, and 24.3° are indexed to the MFI structure, meaning the addition of TEPA into the synthesis gel does not transform the phase texture of the TS-1 under hydrothermal synthesis or calcination. However, the peak intensities have been affected. He et al. [21] reported that the diffraction peak intensity increases with an increase in crystallinity. Mn–Fe/TS-1(R-0.5), Mn–Fe/TS-1(R-1), and Mn–Fe/TS-1(R-2) display lower crystallinity than Mn–Fe/TS-1(R-0), ascribed to the existence of small crystallites [22]. Furthermore, the crystalline phases of Mn and Fe species are not detected in Mn–Fe/TS-1(R-*x*) catalysts, indicating that the particle size of Mn and Fe species is too small to be detected; the Mn and Fe species are amorphous [23].

### 2.2. FT−IR Spectroscopy

**Figure 2:**
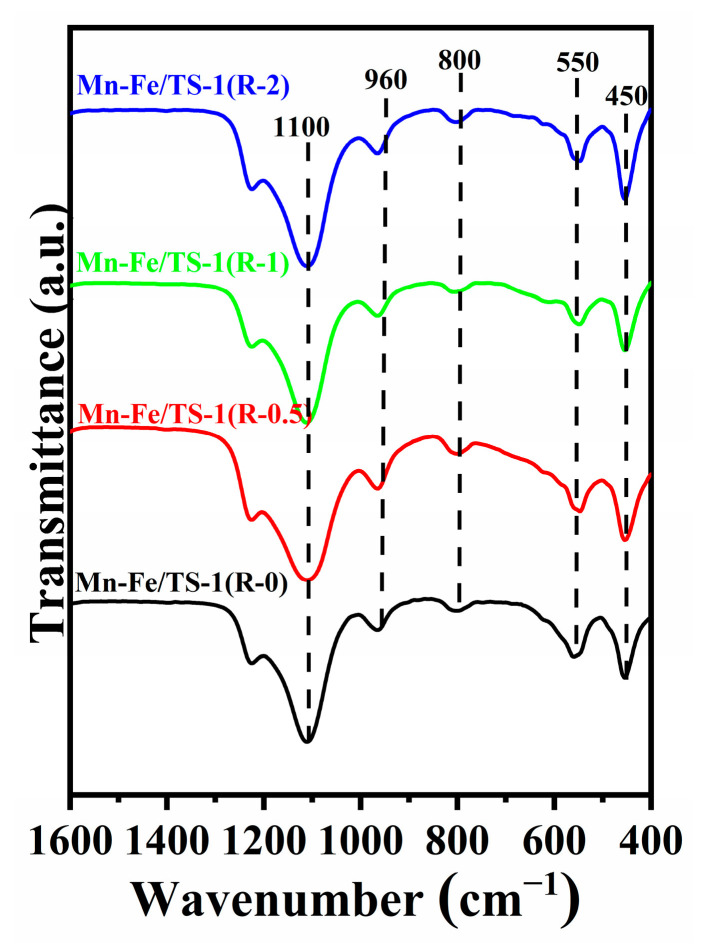
FT−IR spectra of Mn–Fe/TS-1(R-0), Mn–Fe/TS-1(R-0.5), Mn–Fe/TS-1(R-1), and Mn–Fe/TS-1(R-2).

The FT−IR spectra of Mn–Fe/TS-1(R-0), Mn–Fe/TS-1(R-0.5), Mn–Fe/TS-1(R-1), and Mn–Fe/TS-1(R-2) are shown in Figure 2. All samples display infrared peaks at 1100, 960, 800, 550, and 450 cm^−1^. The band at 550 cm^−1^ is assigned to the vibration of the double five-membered ring unit and demonstrates the formation of MFI structure. The bands at 800 and 1100 cm^−1^ are attributed to the symmetrical and antisymmetrical stretching vibrations of Si–O–Si bonds, respectively. The band at 960 cm^−1^ has been used as evidence of the isomorphous substitution of Ti in the TS-1 framework [24]. Moreover, the intensity ratio of the bands at 550 and 450 cm^−1^ (I_550_/I_450_) has been often used to evaluate the crystallinity of MFI zeolite, which is termed as the FTIR crystallinity [25,26]. The I_550_/I_450_ ratios for the Mn–Fe/TS-1(R-0), Mn–Fe/TS-1(R-0.5), Mn–Fe/TS-1(R-1), and Mn–Fe/TS-1(R-2) are 0.66, 0.52, 0.59, and 0.54, respectively, demonstrating that Mn–Fe/TS-1 synthesized using TEPA as a metal complexing agent has low crystallinity, which is in good agreement with the XRD results.

### 2.3. N_2_ Adsorption–Desorption

**Figure 3:**
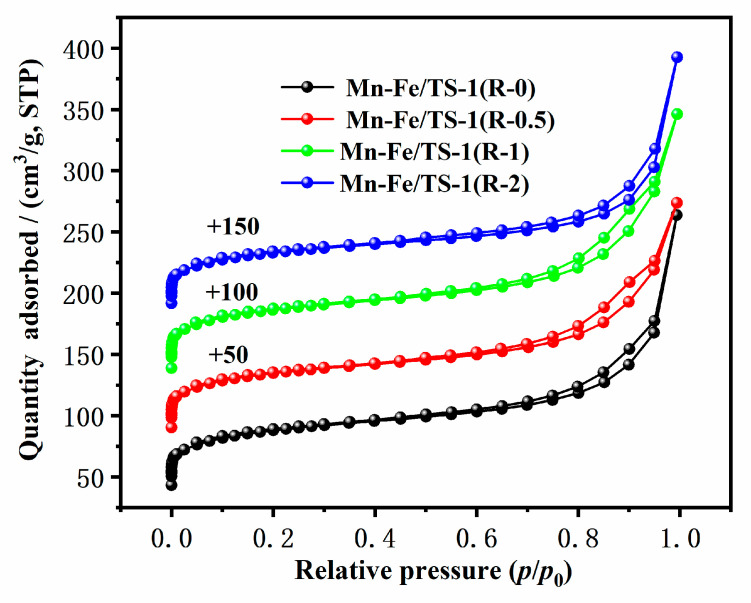
N_2_ adsorption–desorption isotherms of Mn–Fe/TS-1(R-0), Mn–Fe/TS-1(R-0.5), Mn–Fe/TS-1(R-1), and Mn–Fe/TS-1(R-2).

The N_2_ adsorption–desorption isotherms of Mn–Fe/TS-1(R-0), Mn–Fe/TS-1(R-0.5), Mn–Fe/TS-1(R-1), and Mn–Fe/TS-1(R-2) are presented in Figure 3 and the textural properties of the catalysts are presented in Table 1. All of the Mn–Fe/TS-1(R-*x*) samples displayed standard type I isotherms in the relative pressures of p/p_0_ < 0.01, indicating that the samples have microporous structure. Meanwhile, an uptake in the relative pressures of 0.60 < p/p_0_ < 1.0 can be observed due to the intercrystalline mesopores, which is typical of the nanocrystal structure of TS-1 [27]. The hierarchical structure of the samples is conducive to enhancing the diffusion of reactant and product molecules [28]. Moreover, the Mn–Fe/TS-1(R-2) catalyst displays a higher content of active metal components (3.9 wt% Mn and 4.9 wt% Fe) than Mn–Fe/TS-1(R-0, 0.5 wt% Mn and 2 wt% Fe), which demonstrates that the suitable addition of TEPA is conducive to increasing a number of active components. Significantly, high levels of Mn and Fe in Mn–Fe/TiO_2_ prepared by the impregnation method can be observed, which promote most of the active ingredient loaded on the support and reduce the level of the active ingredient dispersion.

### 2.4. SEM Images and EDS Analysis

**Figure 4:**
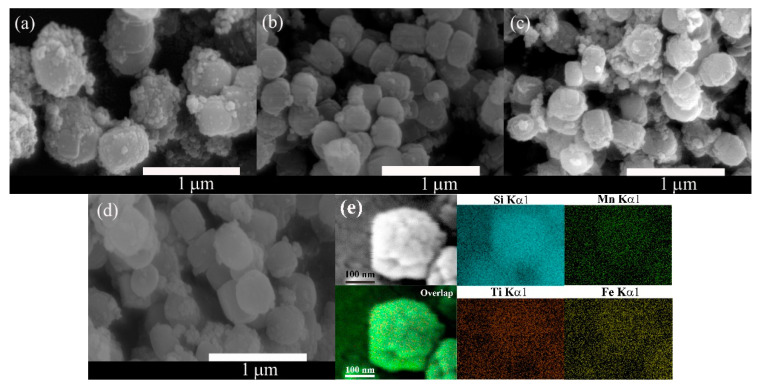
SEM images of Mn–Fe/TS-1(R-0) (**a**), Mn–Fe/TS-1(R-0.5) (**b**), Mn–Fe/TS-1(R-1) (**c**), Mn–Fe/TS-1(R-2) (**d**), and EDS mapping results of Mn–Fe/TS-1(R-2) (**e**).

The morphology and particle size of Mn–Fe/TS-1(R-*x*) were characterized by SEM, as shown in Figure 4. The calcined Mn–Fe/TS-1(R-*x*) shows granular morphology with a rough surface. The particle size of Mn–Fe/TS-1(R-0) is 400–700 nm (Figure 4a). Interestingly, with the introduction of a small amount of TEPA for Mn–Fe/TS-1(R-0.5), the crystal size reduces to 200–350 nm (Figure 4b). However, upon further increasing the TEPA amount, the crystal size significantly increases again from 270–400 nm to 300–450 nm for Mn–Fe/TS-1(R-1) (Figure 4c) and Mn–Fe/TS-1(R-2) (Figure 4d), respectively. The results show that the addition of TEPA does not restrain the generation of TS-1, but influences the crystallinity of TS-1 to a certain extent in accordance with the XRD results. The EDX mapping of the Mn–Fe/TS-1(R-2) (Figure 4e) indicates that excluding Si and Ti, which constitute the framework of TS-1, Mn and Fe are also detected in the crystallite. Therefore, Mn and Fe species may be incorporated into the structure of the skeleton or cationic sites and may be highly dispersed over the catalyst [29].

### 2.5. XPS Analysis

**Figure 5:**
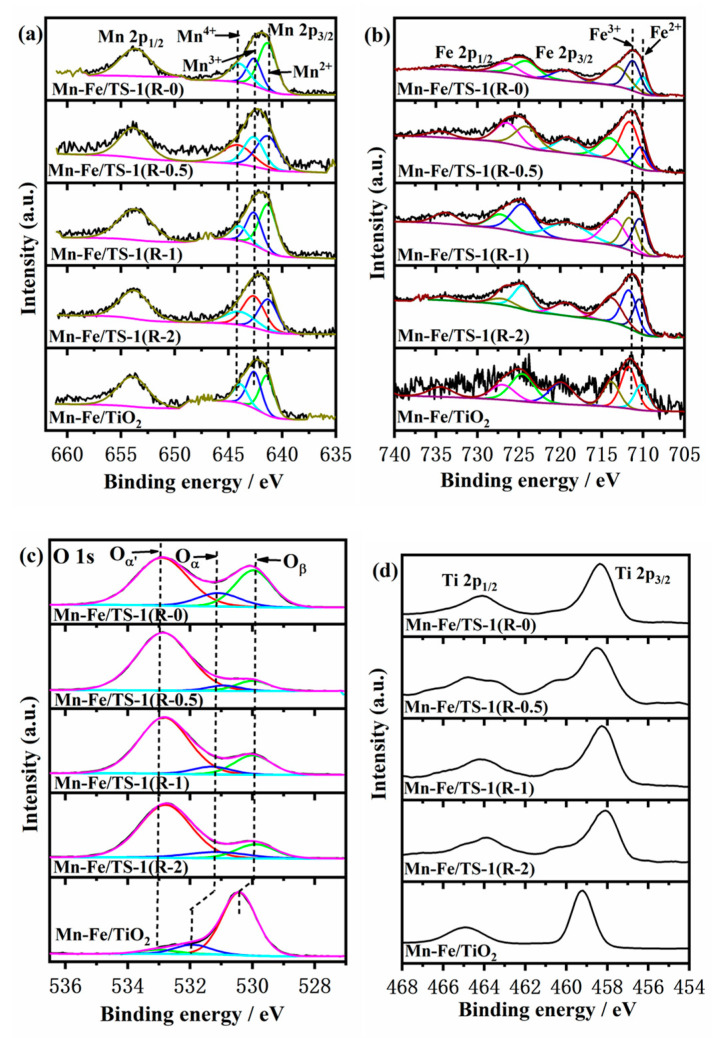
Mn 2p (**a**), Fe 2p (**b**), O 1s (**c**), and Ti 2p (**d**) XPS spectra.

The surface composition and chemical state of Mn, Fe, O, and Ti of the different catalysts were characterized by XPS (Figure 5). As shown in Figure 5a, the Mn 2p XPS spectra exhibit two main peaks, associated with Mn 2p_1/2_ (~653 eV) and Mn 2p_3/2_ (~642 eV). The Mn 2p_3/2_ spectra of the catalysts are de-convoluted into oxidation states of Mn^2+^, Mn^3+^, and Mn^4+^ which are observed at 641.3, 642.5, and 644.2 eV, respectively. Moreover, the relative ratios of Mn^4+^/Mn_suf_ for all the catalysts were calculated and the results are listed in Table 2. The Mn^4+^/Mn_suf_ values of Mn–Fe/TS-1(R-0.5) and Mn–Fe/TS-1(R-2) are significantly higher than that of others. Combined with the denitration results of the catalysts, the greater Mn^4+^ can accelerate the transformation of NO to NO_2_ and further promote the occurrence of a “fast SCR” reaction [30]. 

The Fe 2p XPS spectra are shown in Figure 5b, and display two main peaks of Fe 2p_1/2_ and Fe 2p_3/2_. The Fe 2p_3/2_ peak is deconvoluted into different states of Fe consisting of Fe^2+^ and Fe^3+^ species, which appear at 710 eV and 711 eV [31], respectively. The relative ratio of Fe^2+^/Fe_suf_ is increased from 8.71% (Mn–Fe/TS-1(R-0)) to 15.8% (Mn–Fe/TS-1(R-2)) with the increased addition of TEPA. Compared with Mn–Fe/TiO_2_ catalyst prepared by the wet impregnation method, the relative ratio of Fe^2+^/Fe_suf_ over the Mn–Fe/TS-1(R-2) catalyst is higher. Therefore, the Mn–Fe/TS-1(R-2) catalyst has more active sites to accelerate the SCR reaction. The atomic concentration of the Mn–Fe/TS-1(R-2) (0.21 % Mn, 3.43 % Fe, Table 2) catalyst is lower than that of Mn–Fe/TS-1(R-0, 0.5, 1), which demonstrates that the majority of the active metal component on the surface is in the form of Mn^4+^ and Fe^2+^.

The O 1 s spectra of the catalysts are deconvoluted into three peaks as shown in Figure 5c, corresponding to the lattice oxygen (represented by O_β_) at around 530 eV, surface chemisorbed oxygen (represented by O_α_) at around 531 eV, and –OH (represented by O_α’_) at around 532.8 eV. The surface chemisorbed oxygen is an extremely active oxygen species that plays a key role in oxidation reactions attributed to more rapid migration than the other oxygen species. Therefore, the high O_α_/O_suf_ atomic ratio is conducive to accelerating the transformation of NO to NO_2_ to improve the NH_3_-SCR reaction performance. The relative ratio of O_α_/O_suf_ decreases in the following order: Mn–Fe/TS-1(R-0) > Mn–Fe/TS-1(R-2) > Mn–Fe/TS-1(R-1) > Mn–Fe/TiO_2_> Mn–Fe/TS-1(R-0.5). Furthermore, the O_α’_ peak intensity of Mn–Fe/TS-1(R-*x*) catalysts is stronger than that of Mn–Fe/TiO_2_. The results illustrate that Mn–Fe/TS-1(R-*x*) catalysts possess more –OH than the Mn–Fe/TiO_2_ catalyst, which is mainly derived from the Si–OH and Ti–OH of the TS-1 support [31].

The Ti 2p XPS spectra of all catalysts show two main peaks as shown in Figure 5d, associated with Ti 2p_1/2_ (~464.3 eV) and Ti 2p_3/2_ (~458.3 eV). The results indicate that Ti^4+^ is the main valence state of all catalysts [32]. The sectional Ti atoms in the TiO_2_ support are replaced by Si, and the binding energy of the Mn–Fe/TS-1(R-*x*) catalyst shifts to a high value, indicating that the introduction of Si affects the chemical environment of Ti^4+^ in the catalyst.

### 2.6. H_2_-TPR 

**Figure 6:**
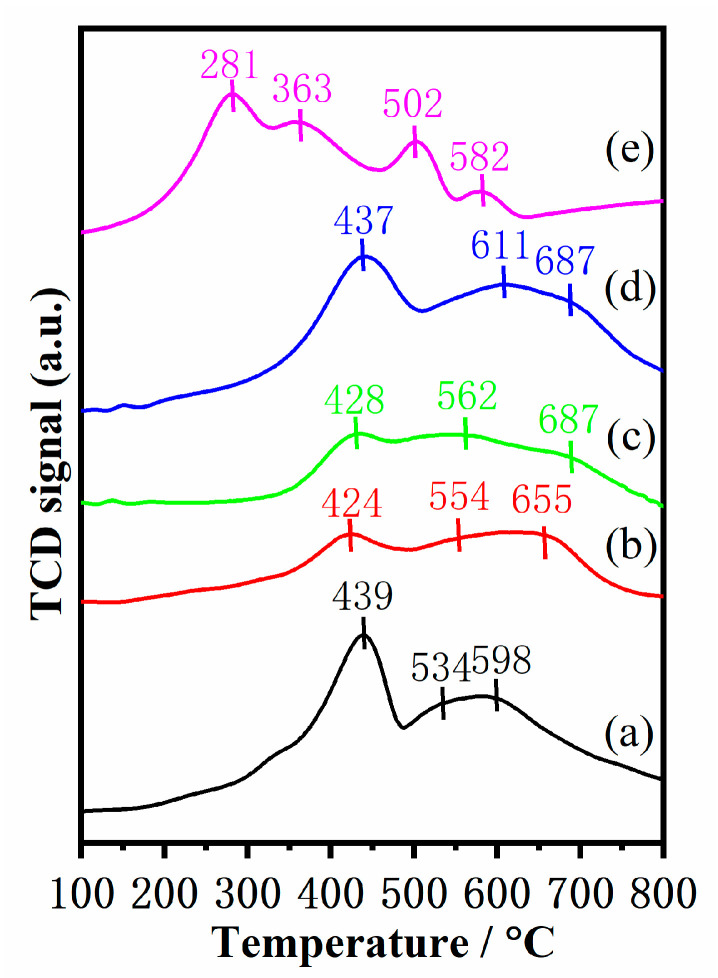
H_2_-TPR profiles of (**a**) Mn–Fe/TS-1(R-0), (**b**) Mn–Fe/TS-1(R-0.5), (**c**) Mn–Fe/TS-1(R-1), (**d**) Mn–Fe/TS-1(R-2), and (**e**) Mn–Fe/TiO_2_.

The reducibility of catalysts closely correlates with the catalytic performance of the NH_3_-SCR reaction. Hence, H_2_-TPR experiments were carried out to characterize the reducibility of the catalysts and the result are displayed in Figure 6. The H_2_ consumption peaks are observed from 100 to 800 °C in all catalysts, which are related to the reduction process of MnO*_x_* and FeO*_x_*. Mn–Fe/TiO_2_ catalyst exhibits four obvious reduction peaks. The first peak at low temperatures (~281 °C) is assigned to the reduction of MnO_2_ to Mn_2_O_3_. The second reduction peak at around 363 °C is attributed to the reduction of Mn_2_O_3_ to Mn_3_O_4_ and Fe_2_O_3_ to Fe_3_O_4_. This reduction process is more liable to happen over reducible sites in the form of oligomeric clusters, nanoparticles, or isolated ions, while residual Mn_2_O_3_ and Fe_2_O_3_ reducing to MnO and Fe_3_O_4_ mostly occurs at relatively higher temperatures (Peak 3, 502 °C). The fourth reduction peak at high temperatures (~582 °C) belongs to the overlapping peak of Mn_3_O_4_→MnO and Fe_3_O_4_→FeO [33]. The results indicate that most Fe_2_O_3_ is reduced at lower temperatures (~363 °C), while only small amounts of remaining Fe_2_O_3_ are reduced to Fe_3_O_4_ at higher temperatures. Three obvious reduction peaks are observed in the Mn–Fe/TS-1(R-*x*) catalysts. The reduction peaks at around 430 °C could be ascribed to the stepwise reduction of MnO_2_ and Fe_2_O_3_ (MnO_2_→Mn_2_O_3_, Mn_2_O_3_→Mn_3_O_4_, and Fe_2_O_3_→ FeO). The reduction peak located at 530–610 °C is associated with the reduction of Mn_3_O_4_, while the high-temperature reduction peaks (590–690 °C) are related to the reduction of FeO [34]. When Si species were introduced into the TiO_2_ support, H_2_ consumption of the Mn–Fe/TS-1(R-*x*) catalysts was larger than that of the Mn–Fe/TiO_2_ catalyst (Table 3). It is worth noting that the Mn–Fe/TS-1(R-2) catalyst displays higher H_2_ consumption than the others, indicating that it possesses enhanced redox properties. The improved reducibility is beneficial to promote the NH_3_-SCR reaction.

### 2.7. NH_3_-TPD 

**Figure 7:**
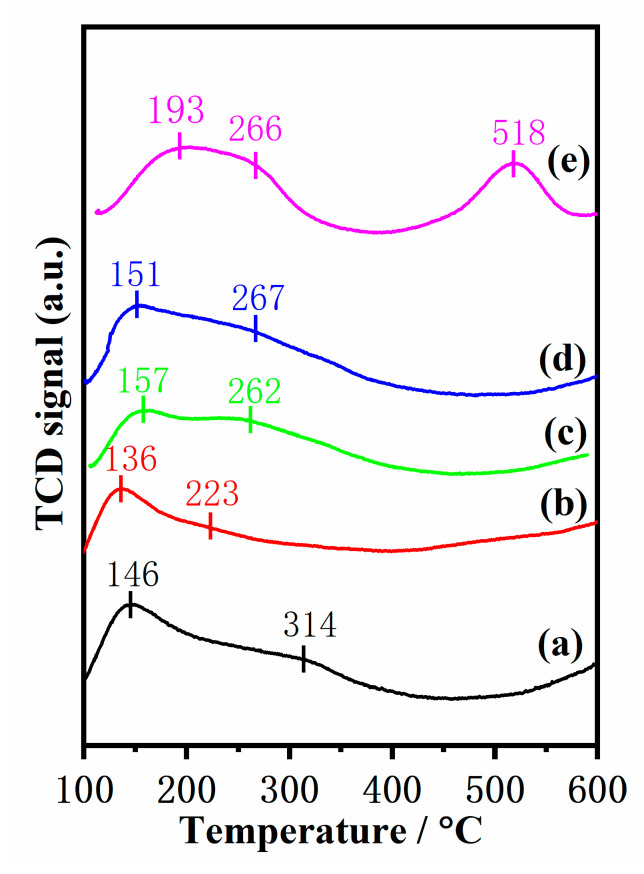
NH_3_-TPD profiles of (**a**) Mn–Fe/TS-1(R-0), (**b**) Mn–Fe/TS-1(R-0.5), (**c**) Mn–Fe/TS-1(R-1), (**d**) Mn–Fe/TS-1(R-2), and (**e**) Mn–Fe/TiO_2_.

The catalyst surface acidity is a very crucial influencing factor in low-temperature SCR reactions, and the acidity of the catalysts was determined by NH_3_-TPD. According to previous studies [35,36], the coordinated NH_3_ molecules bound to Lewis acid sites is more thermally stable than the NH_4_^+^ ions fixed on Brønsted acid sites, so it could be conjectured that the desorption peak at low temperatures is assigned to NH_4_^+^ ions bound to the Brønsted acid sites, while the desorption peak at high temperatures is associated with NH_3_ molecules originating from the Lewis acid sites. Moreover, the area of desorption peaks is directly proportional to the acid amount and the peak position is correlated with the acid strength. As shown in Figure 7, three ammonia desorption peaks are discovered in the Mn–Fe/TiO_2_ catalyst; the desorption peak at low temperatures (~193 °C) is generated by the physical adsorption of NH_3_, the desorption peak at 200–300 °C is attributed to the Brønsted acid site, and the desorption peak at high temperatures (~518 °C) is attributed to the Lewis acid site [34]. It is worth noting that Mn–Fe/TS-1(R-x) catalysts display two desorption peaks. The desorption peak at low temperatures (<200 °C) is attributed to the physical adsorption of NH_3_, and the peak at high temperatures (200–400 °C) is ascribed to the Brønsted acid site [37]. Previous research indicates that the Brønsted acid site could reserve NH_3_ and enhance SCR reaction activity [38,39]. The amounts of different acid sites are calculated from the NH_3_-TPD results. As listed in Table 4, the Brønsted acid amount of Mn–Fe/TS-1(R-2) (centered at around 267 °C) is higher than those of others catalysts, which is conducive to improving the SCR reaction, indicating that the substitution of Si species into the TiO_2_ support can enhance the surface acidity of the catalyst and the adsorption and activation of ammonia, thus improving the catalytic activity in low-temperature SCR reactions. The acidic properties of the catalysts were also analyzed by pyridine IR spectroscopy (Figure S1 in Supplementary Materials). IR bands at ~1445 and 1540 cm ^−1^ observed in the spectra can be attributed to pyridine adsorption related to Lewis and Brønsted acid sites, respectively. The Mn–Fe/TS-1(R-2) exhibits a higher peak area of Brønsted acid sites than the other samples, indicating that the amount of Brønsted acid sites on Mn–Fe/TS-1(R-2) catalyst was significantly increased compared with the other catalysts. This is consistent with the NH_3_-TPD result.

### 2.8. NH_3_-SCR Performance

**Figure 8:**
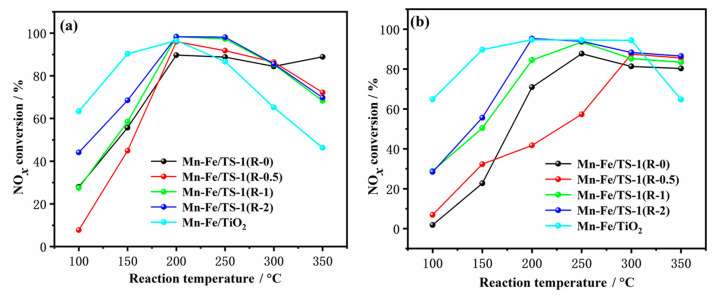
NO*_x_* conversion as a function of reaction temperatures over the Mn–Fe/TS-1(R-0), Mn–Fe/TS-1(R-0.5), Mn–Fe/TS-1(R-1), Mn–Fe/TS-1(R-2), and Mn–Fe/TiO_2_ in the absence (**a**) and presence of H_2_O and SO_2_ (**b**). Reaction conditions: 500 ppm NO, 500 ppm NH_3_, 5% O_2_, 100 ppm SO_2_ (when used), 5 vol % H_2_O (when used), and balanced with N_2_; the total flow rate was 100 mL/min.

Figure 8a displays NO*_x_* conversion as a function of reaction temperature over Mn–Fe/TS-1(R-0), Mn–Fe/TS-1(R-0.5), Mn–Fe/TS-1(R-1), and Mn–Fe/TS-1(R-2) catalysts with different amounts of added TEPA. To study the influence of TS-1 support on NH_3_-SCR reactions, the NO*_x_* conversion over the Mn–Fe/TiO_2_ catalyst prepared by the wet impregnation method was also evaluated. Mn–Fe/TiO_2_ and Mn–Fe/TS-1(R-2) show higher NO*_x_* conversion than other catalysts at low temperatures (<200 °C). However, the NO*_x_* conversion of Mn–Fe/TiO_2_ decreases due to the generation of N_2_O and NO_2_ byproducts at high temperatures (when the reaction temperature increases above 250 °C) [14]. Furthermore, TiO_2_ support undergoes phase transition at high temperatures (>550 °C), leading to narrow temperature windows for SCR reactions. In contrast, the NH_3_-SCR activity of Mn–Fe/TS-1(R-*x*) catalysts is maintained well and only a slight decline in NO*_x_* conversion is observed at high temperatures (>250 °C) due to high thermal stability and the enhanced acidity of TS-1 support. It is worth noting that the Mn–Fe/TS-1(R-2) catalyst exhibits remarkably improved catalytic activity with more than 80% NO*_x_* conversion in a wide temperature range of 170–325 °C. Furthermore, Mn–Fe/TS-1 catalysts synthesized using the wet impregnation method were reported by Wang et al. [20], demonstrating that the optimized Mn_3_–Fe_2_/TS-1-30 can maintain steady NO*_x_* conversion efficiencies above 80% in the temperature range of 110–230 °C with a space velocity of 18,000 h^−1^. Meanwhile, Mn–Fe/TS-1(R-2) prepared by the one-pot hydrothermal method displays wider temperature ranges with high GHSV than the Mn_3_–Fe_2_/TS-1-30 catalyst.

The resistance to H_2_O and SO_2_ poisoning was further evaluated over the Mn–Fe/TS-1(R-*x*) and Mn–Fe/TiO_2_ catalysts, and the results are shown in Figure 8b. Previous research results show that H_2_O and SO_2_ combine with NH_3_ to produce NH_4_HSO_4_ with the coexistence of H_2_O and SO_2_ [28,40], and the NH_4_HSO_4_ cannot decompose below 300 °C [41]. As can be seen in Figure 8b, the NO*_x_* conversion of Mn–Fe/TS-1(R-*x*) markedly decreases with the introduction of H_2_O and SO_2_ at low temperatures (≤300 °C). Conversely, the inhibition effect on the NH_3_-SCR activity of Mn–Fe/TiO_2_ is less than that of Mn–Fe/TS-1(R-*x*) in the presence of H_2_O and SO_2_. More than 80% NO*_x_* conversion is achieved over the Mn–Fe/TiO_2_ catalyst at 150–300 °C, which may be ascribed to the regeneration of the acid site from NH_4_HSO_4_ on the catalyst surface. When the temperature reaches 350 °C, the NO*_x_* conversion of the Mn–Fe/TiO_2_ catalyst decreases to 65% due to the decomposition of NH_4_HSO_4_ covered on the surface at high temperatures (>300 °C). It is important to note that the NO*_x_* conversion of the Mn–Fe/TS-1(R-2) catalyst reaches a value above 80% at temperatures above 200 °C, indicating that the catalytic activity is less affected by the introduction of H_2_O and SO_2_. The enhanced H_2_O and SO_2_ tolerance obtained with Mn–Fe/TS-1(R-2) may be due to the addition of a suitable amount of complexing agent TEPA, which facilitates more active component loading of the TS-1 support and a micro-mesoporous structure beneficial for the adsorption and diffusion of the reactants and products. Based on the above results, titanosilicate TS-1 support with desirable pore structure and enriched isolated framework Ti species can enhance SCR activity. It is important to point out that SSZ-13 has been commonly used in NH_3_-SCR reactions due to its unique pore configuration and acidity properties. However, tetra-coordinated titanium-incorporated SSZ-13 zeolites show stronger Brønsted acidity and hydrothermal stability than SSZ-13 [42], indicating them as a promising SCR catalyst in the future.

## 3. Materials and Methods

### 3.1. Catalyst Preparation

The precursors of the silicon source and titanium source were tetraethylorthosilicate (99%, TEOS, innochem) and tetrabutyl orthotitanate (98%, TBOT, Aladdin), respectively. Tetraethylenepentamine (TEPA, Aladdin) was used as the complexing agent, and tetrapropyl ammonium hydroxide (25%, TPAOH, innochem) was employed as a structure-directing agent. Manganese nitrate (Mn(NO_3_)_2_•4H_2_O, Merck) and iron nitrate (Fe(NO_3_)_3_•9H_2_O, Aladdin) were employed as metal sources.

The crystal seed of TS-1 was prepared with a gel composition of 5 SiO_2_:0.1665 TiO_2_:1.5 TPAOH: 150 H_2_O through the following steps: First, 5.63 mL TEOS and 0.28 mL TBOT were mixed well to obtain Solution A. Later, 6.0 mL TPAOH was dissolved in 13.5 mL water to form Solution B. Solution A mixed with Solution B and stirred for 10 h to achieve a uniform gel. The resulting gel was poured into a stainless-steel autoclave for hydrothermal crystallization at 200 °C for 8 h to obtain the crystal seed of TS-1. An amount of 1.27 g Mn(NO_3_)_2_•4H_2_O and 2.04 g Fe(NO_3_)_3_•9H_2_O were dissolved in 8.5 mL water, then different amounts of TEPA (0, 0.48, 0.96, 1.92 mL) were added in the solution dropwise and stirred for 1 h. Subsequently, the crystal seed of TS-1 was added to the above solution and stirred for 1 h to acquire a uniform mixture. Then, the obtained mixture was transferred to an autoclave and statically crystallized for 40 h at 200 °C. The collected precipitate was filtered, washed with distilled water, and dried at 60 °C overnight. Finally, the product was calcinated in an air flow at 550 °C for 6 h. The molar composition of the catalyst was: 5 SiO_2_: 0.1665 TiO_2_: 1.5 TPAOH: 150 H_2_O: 1.0 Mn(NO_3_)_2_•4H_2_O: 1.0 Fe(NO_3_)_3_•9H_2_O: *x* TEPA. The product was named Mn–Fe/TS-1(R-*x*), where *x* denotes the mole ratios of TEPA/Mn (*x* = 0, 0.5, 1, and 2).

For comparison, Mn–Fe/TiO_2_ was also synthesized by an impregnation method. A total of 2.04 g Fe(NO_3_)_3_•9H_2_O and 1.27 g Mn(NO_3_)_2_•4H_2_O were dissolved in 20 mL water, then 2.1 g TiO_2_ (P25) was impregnated into the above solution. The resulting mixture was dried in a water bath at 80 °C to obtain a powder, and calcined under air at 500 °C for 5 h to obtain the Mn–Fe/TiO_2_ catalyst.

### 3.2. Catalyst Characterization

A Shimadzu XRD-6000 diffractometer (Shimadzu, Japan) was used to explore the crystal phase using Cu Kα radiation (λ = 0.15418 nm). Fourier transform infrared (FT-IR) spectra of the samples were recorded on a Spectrum 1 spectrometer (PE, Waltham, Mass, USA) using KBr disks. The textural properties of catalysts were investigated using N_2_ adsorption–desorption isotherms at 77 K on a Micromeritics ASAP 2460 (Micromeritics, Norcross, Ga, USA). The total surface area (S_BET_) was calculated based on the BET formula. The mesopore surface area (S_meso_), micropore volume (V_micro_), and mesopore volume (V_meso_) were determined by the t-plot method. The active metal contents of the catalysts were measured by an Agilent 7500Ce (Agilent, Santa Clara, California, USA) using inductively coupled plasma mass spectrometry (ICP-MS). Morphologies and particle sizes were measured using a scanning electron microscope (Hitachi S-4300) equipped with an energy dispersive spectrometer (EDS) for analyzing the dispersion of metal oxides. Nano Measurer software (China) was used to analyze the size of crystal particles. At least 300 particles per sample were measured for confirming the average particle size. XPS spectroscopy was conducted using a Thermo ESCALAB 250Xi spectrometer (Thermo, Waltham, MA, USA) equipped with a monochromatized Al Ka X-ray source (1486.6 eV). C 1s (binding energy 284.8 eV) served as a reference. Temperature-programmed reduction with hydrogen (H_2_-TPR) was performed on a Micromeritics ChemiSorb 2720 analyzer. A 0.1 g sample was pretreated at 400 °C for 1 h under Ar flow, then cooled down to 40 °C. The sample was heated to 900 °C at a rate of 10 °C/min under a flow (30 mL/min) of 10 vol.% H_2_/Ar. NH_3_ temperature-programmed desorption (NH_3_-TPD) was analyzed by the same instruments as those used for H_2_-TPR. The sample (~0.1 g) was preheated in a pure Ar stream (30 mL/min) at 500 °C for 1 h and then cooled to 110 °C. The catalysts were pre-treated in 10 % NH_3_/He for 1 h, followed by Ar purging for 1 h. NH_3_ desorption was measured at a ramp of 10 °C min^−1^ in an Ar flow (30 mL/min) from 110 to 700 °C. The pyridine IR spectra were recorded on a Spectrum 1 spectrometer (PE, USA). The 0.02 g samples were saturated and adsorbed by pyridine at 298 K for 30 min after activization at 773 K for 1 h, and then evacuated at 373 K for 1 h.

### 3.3. Catalytic Test

The NH_3_-SCR activity was measured in a fixed-bed quartz flow tube reactor loaded with a 0.3 g catalyst. The composition of reactant gas was 500 ppm NO, 500 ppm NH_3_, 100 ppm SO_2_ (when used), 5 vol % O_2_, 5 vol % H_2_O (when used), and balance N_2_. The total gas flow rate was 100 mL/min and the corresponding gas hourly space velocity (GHSV) was 20,000 mL·g^−1^·h^−1^. The concentration data of NO and NO_2_ were monitored using an MRU OPTIMA7 flue gas analyzer. The NO*_x_* conversion of the catalyst at the steady state was calculated as follows:

$${\mathrm{NO}}_{x}\;\mathrm{conversion}\;[\%]=\frac{{\left[{\mathrm{NO}}_{x}\right]}_{\mathrm{inlet}}-{\left[{\mathrm{NO}}_{x}\right]}_{\mathrm{outlet}}}{{\left[{\mathrm{NO}}_{x}\right]}_{\mathrm{inlet}}}\times 100\;[\%]$$

## 4. Conclusions

A series of Mn–Fe/TS-1(R-*x*) catalysts have been prepared by utilizing TEPA as a metal complexing agent under hydrothermal conditions for NH_3_-SCR reactions. The effects of the addition amounts of TEPA on the structure and catalytic activity of the Mn–Fe/TS-1 catalysts were investigated, showing that the Mn–Fe/TS-1(R-2) catalyst displayed enhanced NH_3_-SCR activity at low temperatures. Moreover, the introduction of H_2_O/SO_2_ had relatively little impact on NO*_x_* conversion for the Mn–Fe/TS-1(R-2) catalyst. In contrast, Mn–Fe/TiO_2_ showed narrow temperature windows for SCR reactions. The introduction of TEPA could improve the dispersion and loading of the Mn^4+^, Fe^2+^, and surface chemisorbed oxygen. Furthermore, Mn–Fe/TS-1(R-2) displayed enhanced reducibility and high Brønsted acid amounts. Therefore, the addition of the appropriate amount of TEPA effectively optimized the structure of the TS-1 support and enhanced the catalytic activity.

## Figures and Tables

**Table 1:** Textural properties and elemental compositions of catalysts.

Samples	*S*_BET_ ^a^ (m^2^/g)	*S*_meso_ ^b^ (m^2^/g)	*V*_total_ ^c^ (cm^3^/g)	*V*_micro_ ^b^ (cm^3^/g)	*V*_meso_ ^b^ (cm^3^/g)	Mn ^d^ (wt %)	Fe ^d^ (wt %)
Mn–Fe/TS-1(R-0)	323	85	0.24	0.10	0.14	3.4	2.1
Mn–Fe/TS-1(R-0.5)	311	83	0.27	0.10	0.17	2.5	1.7
Mn–Fe/TS-1(R-1)	317	82	0.31	0.10	0.21	2.0	1.2
Mn–Fe/TS-1(R-2)	306	74	0.26	0.10	0.16	3.9	4.9
Mn–Fe/TiO_2_	--	--	--	--	--	13.5	17.9

^a^ Calculated using BET method. ^b^ Calculated by the t-plot method. ^c^ Calculated from the adsorption capacity at p/p_0_ of 0.99. ^d^ Calculated using ICP-MS.

**Table 2:** The surface compositions of the obtained samples.

Samples	Atomic Concentration			Atomic Ratio		
	Mn (at. %)	Fe (at. %)	Ti (at. %)	Mn^4+^/Mn_suf_ (%)	Fe^2+^/Fe_suf_ (%)	O_α_/O_suf_ (%)
Mn–Fe/TS-1(R-0)	2.28	6.96	2.58	12.5	8.71	13.0
Mn–Fe/TS-1(R-0.5)	1.46	2.7	1.52	18.7	9.18	4.7
Mn–Fe/TS-1(R-1)	1.88	3.58	1.84	7.3	13.31	7.9
Mn–Fe/TS-1(R-2)	0.21	3.43	1.41	16.1	15.88	8.6
Mn–Fe/TiO_2_	2.43	0.31	22.32	11.4	15.55	7.06

**Table 3:** Reduction temperature peak and H_2_ consumption of the catalysts.

Samples	Temperature (°C)/H_2_ Consumption (mL·g^−1^, STP)				
	Peak 1	Peak 2	Peak 3	Peak 4	Total
Mn-Fe/TS-1(R-0)	439/46.72	534/16.70	598/19.26	--/--	--/82.69
Mn-Fe/TS-1(R-0.5)	424/42.61	554/6.15	655/23.44	--/--	--/72.22
Mn-Fe/TS-1(R-1)	436/5.79	552/36.02	665/5.10	--/--	--/46.91
Mn-Fe/TS-1(R-2)	437/69.74	611/22.46	687/39.23	--/--	--/131.43
Mn-Fe/TiO_2_	281/22.38	363/15.08	502/7.63	582/1.23	--/46.32

**Table 4:** Acid properties of the samples obtained from NH_3_-TPD.

Samples	Temperature (°C)/NH_3_ Adsorption Amount (mL·g^−1^, STP)			
	Peak 1	Peak 2	Peak 3	Total
Mn–Fe/TS-1(R-0)	146/0.10	314/0.15	--/--	--/0.25
Mn–Fe/TS-1(R-0.5)	136/0.07	223/0.11	--/--	--/0.18
Mn–Fe/TS-1(R-1)	157/0.09	262/0.12	--/--	--/0.21
Mn–Fe/TS-1(R-2)	151/0.06	267/0.20	--/--	--/0.26
Mn–Fe/TiO_2_	193/0.07	266/0.16	518/0.10	--/0.33

## Data Availability

Data is available upon reasonable request from the corresponding authors.

## Supplementary Materials

The following supporting information can be downloaded at: www.mdpi.com/10.3390/molecules28073068/s1, Figure S1: Pyridine FT-IR spectra of Mn-Fe/TS-1(R-0) (a), Mn-Fe/TS-1(R-0.5) (b), Mn-Fe/TS-1(R-1) (c), Mn-Fe/TS-1(R-2) (d) and Mn-Fe/TiO_2_ (e) at 373 K.

## Sample Availability

Samples of the catalysts are available from the authors.
