# Supplementary material for: Effect of Metal Complexing on Mn–Fe/TS-1 Catalysts for Selective Catalytic Reduction of NO with NH3
Source: Molecules. 2023 Mar 29;28(7):3068. doi: 10.3390/molecules28073068 (PMC10095777; doi:10.3390/molecules28073068)
Supplement: Supplementary file 1 [file molecules-28-03068-s001.zip › molecules-2248853-supplementary.pdf]

Supplementary Materials

Effect of Metal Complexing on Mn–Fe/TS-1 Catalysts for Selective Catalytic Reduction of NO with NH_3

Yuanyuan Ma [1,*], Wanting Liu [1], Zhifang Li [2], Yuhang Sun [1], Mingyuan Shi [1], Zheng Nan [1], Ruotong Song [1], Liying Wang [1] and Jingqi Guan [3,*]

1 College of Chemistry and Chemical Engineering, Qiqihar University, Qiqihar 161006, China
2 College of Materials Science and Engineering, Qiqihar University, Qiqihar 161006, China
3 Institute of Physical Chemistry, College of Chemistry, Jilin University, 2519 Jiefang Road, Changchun 130021, China
* Correspondence: mayuanyuan1219@126.com (Y.M.); guanjq@jlu.edu.cn (J.G.)

Figure S1

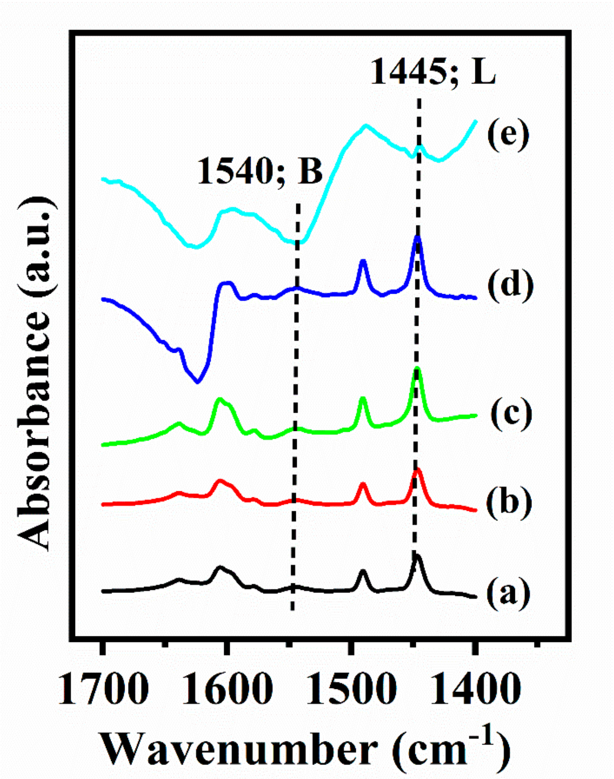

Figure S1. Pyridine FT-IR spectra of Mn-Fe/TS-1(R-0) (**a**), Mn-Fe/TS-1(R-0.5) (**b**), Mn-Fe/TS-1(R-1) (**c**), Mn-Fe/TS-1(R-2) (**d**) and Mn-Fe/TiO_2 (**e**) at 373 K.
